# O070. The association between temperament, depression, hopelessness and quality of life in medication-overuse headache patients

**DOI:** 10.1186/1129-2377-16-S1-A51

**Published:** 2015-09-28

**Authors:** Monica Migliorati, Federica Ricci, Denise Erbuto, Samantha Bellini, Maurizio Pompili

**Affiliations:** Department of Neurosciences Suicide Prevention Center, Sapienza University of Rome, Rome, Italy

## Background

Migraine is one of the major causes of disability and lost working days worldwide[1]. Medication-overuse headache (MOH) is one of the most common forms of chronic daily headache (CDH)[2], with a prevalence in the adult general population of 1%-2%[3]. MOH is often comorbid with emotional disturbances and disordered personality traits. The aim of the present study was to explore the impact of mental illness among patients with migraine. To evaluated if depression, hopelessness, suicide risk and quality of life were associated with temperament and trauma in MOH patients.

## Materials and methods

Participants were 135 consecutive adult outpatients, of which 113 females (83.7%), admitted to the local Headache Centre of the Sant'Andrea Hospital in Rome, Italy. Inclusion criteria were a diagnosis of MOH, and an age of 18 years or older. Exclusion criteria were comorbidity with major disorders of the CNS, delirium and/or any condition affecting the patient's ability to complete the assessment. The average age of participants was 47.59 (SD=12.01). Patients participated voluntarily in the study, and each subject provided written informed consent. Patients were administered the BDI II, BHS, Q-LES-Q, TEMPS-A, SHSS, and CTQ.

## Results

The results showed that females present higher levels of anxiety (t= 3.392; p: < 001). Good correlations were found between temperament and levels of depression (r: -.650; p: < .01), hopelessness (r: -.573; p: < .05), quality of life (-.282<r>-.105; p <0.01) and with the risk of suicide (r: -.36; p: < .05). Particularly, subjects with suicide ideation showed higher scores on the level of depression, (t= -4.823; p: 001) hopelessness, (t= -4.261; p: 001) and emotional abuse (t= -3.526; p: 001).

## Conclusions

Our data confirm that patients with MOH showed an anxiety-related temperament associated with high levels of depression, hopelessness and suicide risk. Suicide attempts seem to be more frequent in patients suffering from migraine than in the general population, especially in females. Suicidal ideation was associated with higher headache frequency and headache-related disability. The evidence of a possible link between chronic headache and psychiatric disorder is not a recent finding. Back in 1895, the occurring depressive mood, irritability, and anxiety in these patients[4] was described. Our findings indicate that patients with a diagnosis of MOH and migraine have severe hopelessness, and suicide risk. This evidence suggests that psychologic assessment is necessary in patients with MOH, and also that the presence of headache has to be carefully monitored in patients with mental illness.

Written informed consent to publication was obtained from the patient(s).

## Conflict of interest

The authors report no conflicts of interest.
